# Phosphoproteomic Comparison of Four *Eimeria tenella* Life Cycle Stages

**DOI:** 10.3390/ijms222212110

**Published:** 2021-11-09

**Authors:** Xueting Ma, Baohong Liu, Zhenxing Gong, Zigang Qu, Jianping Cai

**Affiliations:** 1 State Key Laboratory of Veterinary Etiological Biology, Key Laboratory of Veterinary Parasitology of Gansu Province, Lanzhou Veterinary Research Institute, Chinese Academy of Agricultural Sciences, Lanzhou 730046, China; maxueting@caas.cn (X.M.); gzx1982leo@163.com (Z.G.); quzigang@caas.cn (Z.Q.); 2 Jiangsu Co-Innovation Center for Prevention and Control of Important Animal Infectious Diseases and Zoonoses, Yangzhou 225009, China

**Keywords:** *Eimeria tenella*, life cycle, phosphoproteomic, differentially expressed phosphoproteins (DEPPs)

## Abstract

Protein phosphorylation is an important post-translational modification (PTM) involved in diverse cellular functions. It is the most prevalent PTM in both *Toxoplasma gondii* and *Plasmodium falciparum*, but its status in *Eimeria tenella* has not been reported. Herein, we performed a comprehensive, quantitative phosphoproteomic profile analysis of four stages of the *E. tenella* life cycle: unsporulated oocysts (USO), partially sporulated (7 h) oocysts (SO7h), sporulated oocysts (SO), and sporozoites (S). A total of 15,247 phosphorylation sites on 9514 phosphopeptides corresponding to 2897 phosphoproteins were identified across the four stages. In addition, 456, 479, and 198 differentially expressed phosphoproteins (DEPPs) were identified in the comparisons SO7h vs. USO, SO vs. SO7h, and S vs. SO, respectively. Gene Ontology (GO) term and Kyoto Encyclopedia of Genes and Genomes (KEGG) pathway enrichment analyses of DEPPs suggested that they were involved in diverse functions. For SO7h vs. USO, DEPPs were mainly involved in cell division, actin cytoskeleton organization, positive regulation of transport, and pyruvate metabolism. For SO vs. SO7h, they were related to the peptide metabolic process, translation, and RNA transport. DEPPs in the S vs. SO comparison were associated with the tricarboxylic acid metabolic process, positive regulation of ATPase activity, and calcium ion binding. Time course sequencing data analysis (TCseq) identified six clusters with similar expression change characteristics related to carbohydrate metabolism, cytoskeleton organization, and calcium ion transport, demonstrating different regulatory profiles across the life cycle of *E. tenella*. The results revealed significant changes in the abundance of phosphoproteins during *E. tenella* development. The findings shed light on the key roles of protein phosphorylation and dephosphorylation in the *E. tenella* life cycle.

## 1. Introduction

Coccidiosis is a damaging parasitic disease in chickens that inflicts huge economic losses on the poultry industry. *Eimeria tenella*, the intracellular protozoan parasite that causes cecal coccidiosis in chickens, belongs to the phylum Apicomplexa. It has a complex developmental life cycle with separate exogenous (sporogony) and endogenous (schizogony and gametogony) phases [1]. In the exogenous phase, unsporulated oocysts (non-infective) are shed in chicken feces and undergo sporogony (sporulation) under appropriate conditions to become infective. After oral ingestion of sporulated oocysts (SO), sporozoites are excysted, and they infect chicken intestinal epithelia, after which *E. tenella* enters an endogenous phase involving schizogony and gametogony. After fertilization (gametogony), unsporulated oocysts are expelled into the environment with feces, and they enter the exogenous phase [2,3].

It is known that *E. tenella* undergoes a complex life cycle involving several stages characterized by distinct physiologies and morphologies. Expressed sequence tags (ESTs) have been identified to assess gene expression in different stages, and gene transcription and expression profiles are known to differ between developmental stages [2,3,4,5,6]. Numerous novel and potentially crucial developmentally regulated factors involved in the *E. tenella* life cycle have been identified via gene transcriptional analyses. Amiruddin et al. (2012) [7] analyzed 433 full-length cDNA sequences derived from the second-generation merozoite transcriptome of *E. tenella* to support drug development, and 863 genes were found to be up-regulated in gametocytes, compared with merozoites and sporozoites, based on RNA sequencing (RNA-seq) analysis [2]. These findings should assist the development of drugs targeting *E. tenella* infection. Furthermore, the abundance of proteins in *E. tenella* life cycle stages, including unsporulated oocysts, sporulated oocysts, sporozoites, and second-generation merozoites, has been analyzed by proteomics [8,9,10], and a large number of novel candidate rhoptry proteins were identified [10]. A series of drug-related proteins were also screened via proteomics analysis, revealing the action and resistance mechanisms of anticoccidial agents [1,11,12].

Although multiple genes and proteins are altered during the *E. tenella* life cycle according to transcriptomic and proteomic data, post-translational modifications (PTMs) also exert a significant influence on development [13]. They can result in dramatic changes in the activity, structure, localization, and stability of proteins involved in many critical biological pathways, without the need to alter transcriptional and translational levels [14]. There are many diverse PTMs in *E. tenella* [13], of which protein phosphorylation, which regulates temporal and spatial changes in protein activities and functions, is among the most common [15]. Previous studies have revealed key roles for phosphorylation in all aspects of parasite biology, including differentiation, development, protein localization, invasion, egress, and toxicity [13,16,17,18,19].

In the present work, a phosphoproteomics analysis was performed on four *E. tenella* developmental stages: unsporulated oocysts (USO), partially sporulated (7 h) oocysts (SO7h), sporulated oocysts (SO), and sporozoites (S). We identified differentially expressed phosphoproteins (DEPPs) between stages, revealing the protein phosphorylation landscape throughout the life cycle. The functions and interaction networks of the DEPPs were assessed by integrated bioinformatics analyses to clarify the mechanisms operating during the *E. tenella* life cycle.

## 2. Results

### 2.1. Identification, Quantification, and Classification of Phosphopeptides

**Figure 1:**
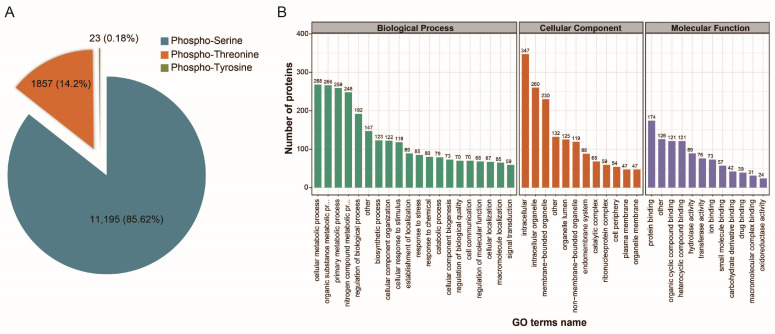
(**A**) Distribution of phosphorylation on serine (pSer), threonine (pThr), and tyrosine (pTyr) for all phosphorylation sites. (**B**) Functional classification of phosphorylated proteins.

In total, 15,247 phosphorylation sites on 9514 phosphopeptides corresponding to 2897 phosphoproteins were identified across the four stages of the *E. tenella* life cycle. Among the phosphorylation sites, phospho-serine (pS) was the most abundant, accounting for 85.62% of all phosphorylated amino acids, while phospho-threonine (pT) accounted for 14.2% and phospho-tyrosine (pY) accounted for 0.18% (Figure 1A). Among the 2897 unique phosphoproteins, 771 were singly phosphorylated, 500 were doubly phosphorylated, 367 were triply phosphorylated, 242 were phosphorylated at four sites, 176 were phosphorylated at five sites, and 841 were phosphorylated at six or more sites. As shown in Figure 1B, phosphorylated proteins were classified into more than 20 groups based on Gene Ontology (GO) terms, including cellular metabolic process, organic substance metabolic process, primary metabolic process, intracellular, intracellular organelle, membrane-bound organelle, protein binding, organic cyclic compound binding, and heterocyclic compound binding.

### 2.2. Identification of Differentially Phosphorylated Proteins

**Figure 2:**
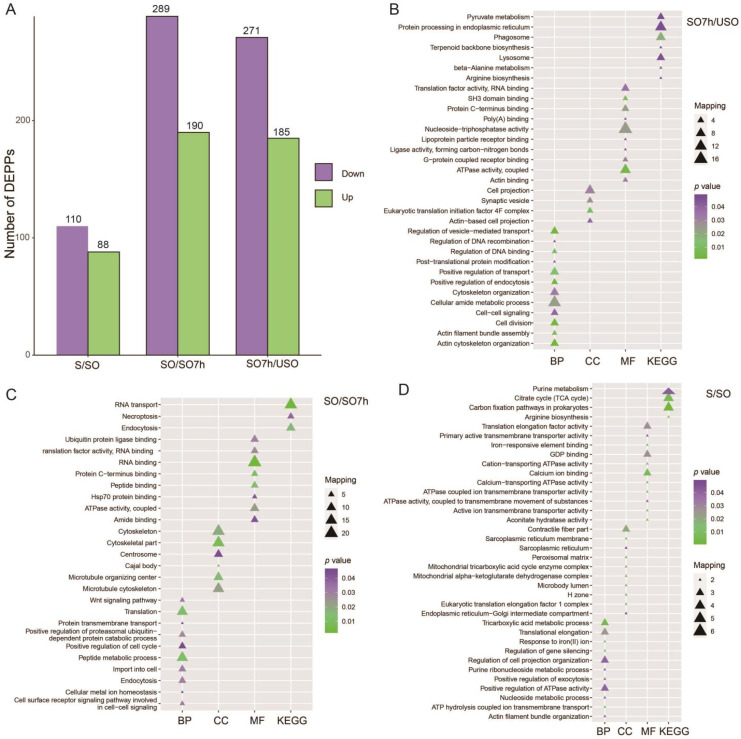
(**A**) The number of DEPPs in each comparison. (**B**) Functional enrichment analysis results for DEPPs in SO7h vs. USO. (**C**) Functional enrichment analysis results for DEPPs in SO vs. SO7h. **(D)** Functional enrichment analysis results for DEPPs in S vs. SO.

DEPPs were identified based on |log2 fold change| >0.58 and *p* < 0.05. A total of 456, 479, and 198 DEPPs were identified from the comparisons SO7h vs. USO, SO vs. SO7h, and S vs. SO, respectively. Among these DEPPs, 271 were up-regulated and 185 were down-regulated for SO7h vs. USO, 190 were up-regulated and 289 were down-regulated for SO vs. SO7h, and 110 were up-regulated and 88 were down-regulated for S vs. SO (Figure 2A).

### 2.3. Functional Enrichment of DEPPs

To better understand the roles of DEPPs in the *E. tenella* life cycle, functional enrichment analysis of GO terms and KEGG pathways was performed on the three groups of DEPPs: SO7h vs. USO, SO vs. SO7h, and S vs. SO, respectively. DEPPs in the SO7h vs. USO comparison were associated with many biological processes such as actin cytoskeleton organization (*p* value = 0.001), including actin (*ETH_00009555*), myosin F (*ETH_00011990*), elongation factor 1 (*ETH_00010290*) and Sec7 domain-containing protein (*ETH_00019375*), cell division (*p* value = 0.004), positive regulation of transport (*p* value = 0.014), and the cellular amide metabolic process (*p* value = 0.02) (Figure 2B). DEPPs in the SO vs. SO7h comparison were linked to the peptide metabolic process (*p* value = 0.012), positive regulation of the proteasomal ubiquitin-dependent protein catabolic process (*p* value = 0.028), endocytosis (*p* value = 0.031), and translation (*p* value = 0.0127) (Figure 2C). For the S vs. SO comparison, DEPPs were associated with multiple biological processes including tricarboxylic acid (TCA) metabolism (*p* value = 0.006) (aconitate hydratase (*ETH_00025665* and *ETH_00020300*)), ATP hydrolysis-coupled ion transmembrane transport (*p* value = 0.014) (calcium-transporting ATPase (*ETH_00002655*) and P-type Ca^2+^-ATPase (*ETH_00006965*)), positive regulation of exocytosis (*p* value = 0.039) (*Rab5c* (*ETH_00021330*) and *Rab2* (*eimer2006e04.tmp10*)), and actin filament bundle organization (*p* value = 0.039) (actin (*ETH_00009555*) and elongation factor 1 (*ETH_00010290*)) (Figure 2D).

KEGG pathway analysis of DEPPs is shown in Figure 2B–D. For SO7h vs. USO, DEPPs were involved in phagosome (*p* value = 0.019), lysosome (*p* value = 0.047), and protein processing in the endoplasmic reticulum (*p* value = 0.0478) (Figure 2B). For SO vs. SO7h, DEPPs were associated with RNA transport (*p* value = 0.002), endocytosis (*p* value = 0.016), and necroptosis (*p* value = 0.038) pathways (Figure 2C). For S vs. SO, DEPPs were linked to citrate (TCA) cycle (*p* value = 0.006) and purine metabolism (*p* value = 0.042) pathways (Figure 2D).

### 2.4. Protein Phosphorylation Patterns

**Figure 3:**
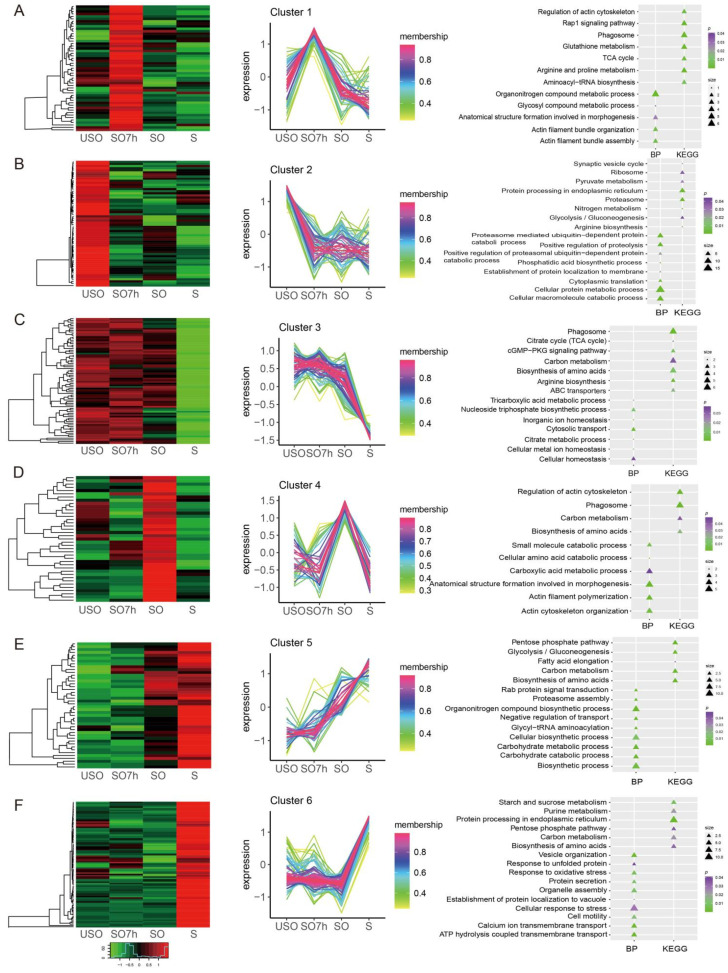
Protein phosphorylation heatmap, time series profiles, and functional enrichment results for (**A**) Cluster1, (**B**) Cluster 2, (**C**) Cluster 3, (**D**) Cluster 4, (**E**) Cluster 5, and (**F**) Cluster 6.

Phosphoproteins were clustered into six profiles using the R package TCseq. Expression levels of phosphoproteins in cluster 1 peaked in SO7h and then constantly decreased at SO and S stages. Proteins in this profile were strongly associated with the organonitrogen compound metabolic process (*p* value = 0.0002), actin filament bundle organization (*p* value = 0.005), and TCA cycle (*p* value = 0.018) subcategories, including aconitate hydratase (*ETH_00020300*) (Figure 3A). There was a clear phosphorylation peak at the USO phase, which then decreased steadily for proteins in cluster 2. Proteins in this profile were linked to protein metabolic processes (*p* value < 0.05) (Figure 3B). Expression levels of phosphoproteins in cluster 3 were steady from USO to SO, and then decreased sharply at the S phase (Figure 3C). Expression levels of phosphoproteins in cluster 4 peaked at the SO phase, and then declined at the S phase (Figure 3D). Expression levels of phosphoproteins in cluster 5 increased dramatically as the life cycle progressed. These proteins were mainly associated with organonitrogen_compound_biosynthetic_processes (*p* value = 0.00047) and the pentose phosphate pathway (*p* value = 2.74 × 10^−^^5^), including transaldolase (*ETH_00011825*) (Figure 3E). Expression levels of phosphoproteins in cluster 6 increased sharply at the S phase. Proteins in this cluster are engaged in calcium_ion_transmembrane_transport (*p* value = 0.00028), protein secretion (*p* value = 0.0097), cell motility (*p* value = 0.014), response to unfolded proteins (*p* value = 0.041) (heat shock protein 40 (*ETH_00029665*), DnaJ domain-containing proteins (*ETH_00006810*)), and the pentose phosphate pathway (*p* value = 0.036) (phosphoglucomutase (*ETH_00002590*), glucose-6-phosphate dehydrogenase (*ETH_00022160*), and transaldolase (*ETH_00011825*)).

### 2.5. Protein–Protein Interaction (PPI) Network Analysis of DEPPs

**Figure 4:**
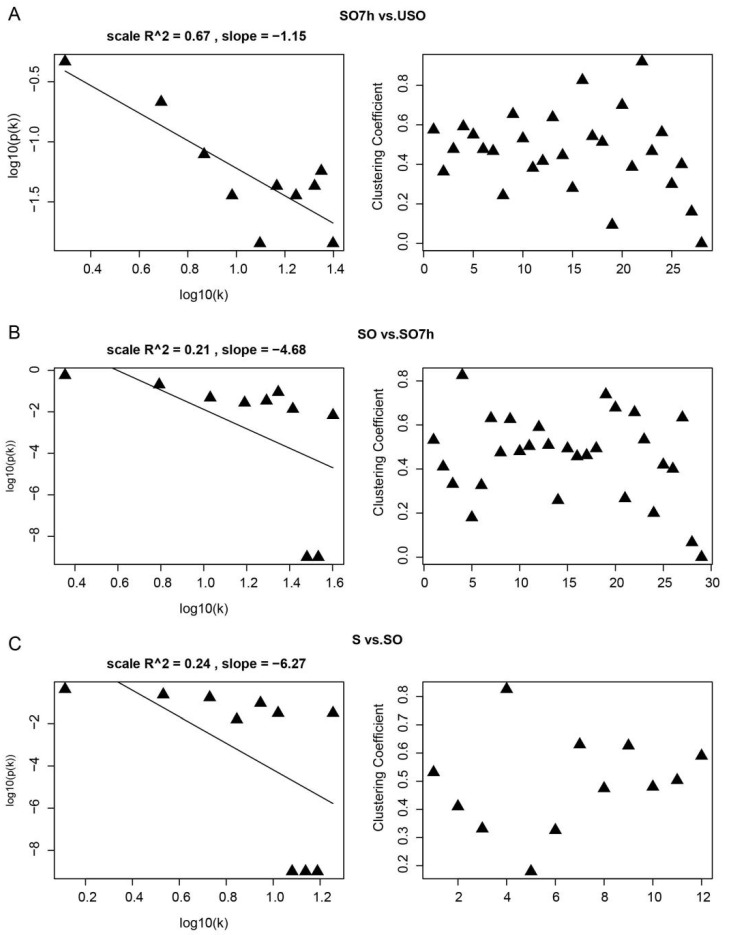
Network topology analysis results for PPI networks based on each comparison. (**A**) SO7h vs. USO. (**B**) SO vs. SO7h. (**C**) S vs. SO.

**Figure 5:**
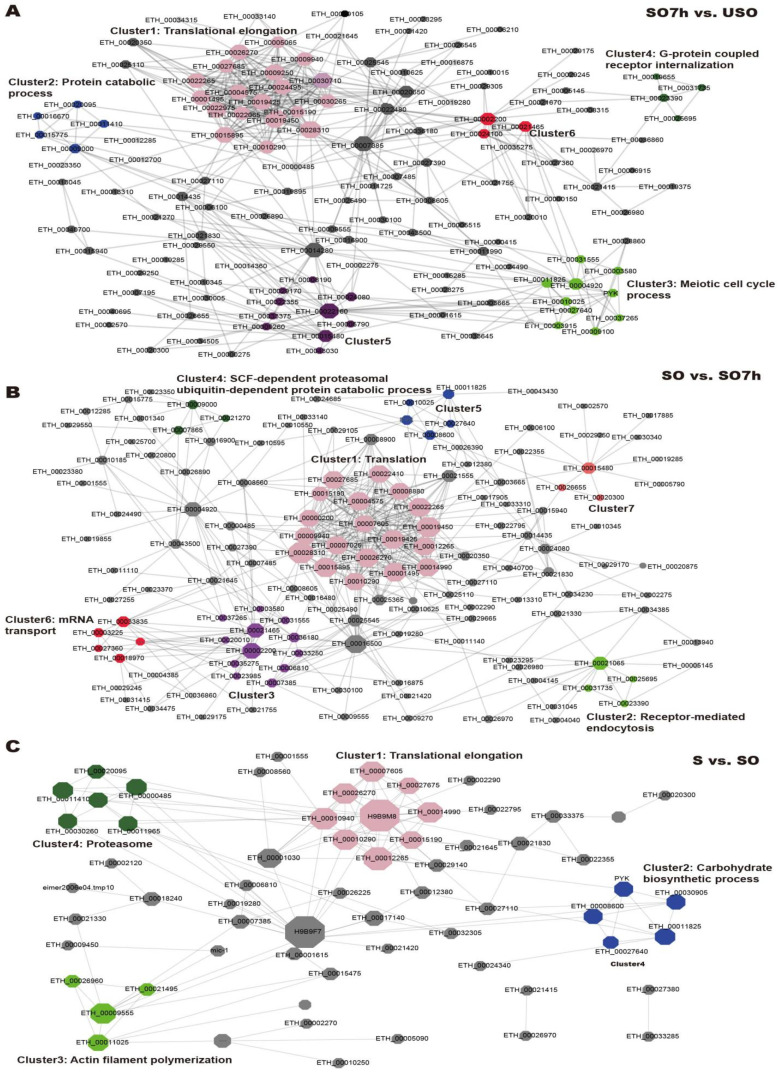
PPI networks for DEPPs based on each comparison. (**A**) SO7h vs. USO. (**B**) SO vs. SO7h. (**C**) S vs. SO. The node size represents the node degree in the PPI network; the nodes in the same module are arranged with the same color and nodes in grey represent proteins which are not clustered in any module using MCODE.

PPI networks were constructed using the STRING database. Network topology analysis showed that all three networks were scale-free and highly modulated (Figure 4). The PPI network for SO7h vs. USO contained 140 proteins and 480 interactions, and six sub-clusters were identified by MCODE. The largest sub-cluster contained 25 proteins involved in translational elongation. The others were associated with the protein catabolic process, meiotic cell cycle process, and G protein-coupled receptor internalization, respectively (Figure 5A). The PPI network for SO vs. SO7h included 147 nodes and 497 edges. The largest sub-cluster was composed of 20 DEPPs engaged in translation. The others were enriched in receptor-mediated endocytosis, meiosis, the SCF-dependent proteasomal ubiquitin-dependent protein catabolic process, and mRNA transport (Figure 5B). There were 64 nodes and 131 edges in the S vs. SO PPI network, with sub-clusters mainly associated with translational elongation, the carbohydrate biosynthetic process, actin filament polymerization, and proteasome (Figure 5C).

## 3. Discussion

It is well acknowledged that carbohydrates play critical roles in parasite life cycles. *E. tenella* possesses multiple carbohydrate-metabolism-related proteins, including many related to the TCA cycle, the pentose phosphate pathway (PPP), gluconeogenesis and glycolysis, and the mannitol cycle, due to its complex life cycle. Metabolic changes are strongly associated with the adaptation of *E. tenella* to aerobic and anaerobic conditions at different development stages.

*E. tenella* contains a TCA-cycle-like pathway that is essential for aerobic respiration [20]. Recent studies reported that the respiration rate is high at the beginning of sporulation but is low when sporulation is complete [21,22]. Wang et al. found that citrate synthase (EtCS), an important TCA cycle enzyme in *E. tenella*, was highly expressed at the unsporulated oocyst stage, suggesting that it may provide energy for parasite development [21]. In this study, we found that DEPPs in S vs. SO were involved in the citrate cycle, and the TCA cycle was enriched in cluster 1, based on TCseq analysis. Phosphorylation levels of these TCA-cycle-related proteins peaked at the SO7h stage and then decreased gradually at the SO and S stages. This trend is consistent with a previous study showing that six enzymes of the TCA cycle were up-regulated during sporulation, including SO, and then down-regulated in sporozoites [20]. In view of this, we speculate that the TCA cycle probably provides much of the energy required for sporulation but is no longer the dominant energy source after oocyst sporulation.

The PPP, including both oxidative and non-oxidative pathways, is involved in producing nucleotide precursors and NADPH [23]. The oxidative arm of the PPP can generate both ribose-5-phosphate (R5P) and NADPH (mode 1). Meanwhile, production of glycolysis can be utilized to provide cells with more R5P than NADPH via the non-oxidative arm (mode 2). In addition, the PPP can channel into glycolysis via its non-oxidative arm to make ATP and NADPH (mode 3) [23]. In *T. gondii*, both the oxidative and non-oxidative arms of the PPP are functional [24,25], and the key enzymes driving carbon from glycolysis to the non-oxidative arm have been identified [25]. In the *P. falciparum* life cycle, the PPP also functions through different modes [23]. For example, the oxidative arm works in the early phase of the parasite life cycle, the non-oxidative arm works via mode 2 in the latter phase, and mode 3 is believed to take effect at the end of the intraerythrocytic life cycle to provide NADPH and ATP [23]. However, little is known about the PPP in *E. tenella*. In this study, we characterized five phosphoproteins involved in the PPP, all of which were up-regulated in sporozoites. For example, glucose-6-phosphate dehydrogenase (G6PD) is a rate-limiting enzyme of the oxidative arm of the PPP [25], while phosphoglucomutase and transaldolase are involved in non-oxidative reactions of the PPP [25]. Phosphoglucomutase converts five-carbon sugars or D-ribose-1-phosphate generated during the salvage of purines into R5P, which can be processed and utilized in glycolysis [23]. Transaldolase is an essential enzyme that produces fructose-6-phosphate (F6P) and erythrose-4-phosphate in the non-oxidative arm, and these compounds are reportedly involved in the oxidative arm of the PPP in *T. gondii* [24,25]. It is crucial that the non-oxidative arm of the PPP connects pentose and hexose monophosphate pools [25]. Based on our results, we speculate that the PPP might play roles in multiple stages of the *E. tenella* life cycle through various modes.

Amylopectin granules are present in sporozoites, sporocyst residual bodies, and merozoites of *E. tenella* [26]. Amylopectin and amylose are the major components of starch. Amylopectin, thought to be the major polysaccharide storage form in *E. tenella*, can be converted into glucose and further into mannitol [27], and is considered the energy source for excystation and the penetration of cells in sporozoites [28]. The key role of amylopectin in the infectivity and viability of *E. tenella* sporozoites has been confirmed [29,30]. Amylopectin is also considered a source of energy for sporulation [27,28]; granules decrease in number and size during sporulation and sporozoite survival, indicating a key role for amylopectin in these two phases [31]. Herein, we identified proteins involved in starch and sucrose metabolism in cluster 6 that were significantly up-regulated at the sporozoite stage, indicating that amylopectin might provide energy at this stage.

Apicomplexan protozoa and other ‘typical’ single-celled eukaryotes share many cytoskeletal elements including microtubules, actin, myosin, and intermediate filament-like proteins [32]. In our study, some cytoskeletal proteins displayed differential phosphorylation levels during the *E. tenella* life cycle. For example, myosin F, previously reported to be conserved within the phylum [33], was significantly up-regulated in the sporozoite phase (in cluster 6). In *T. gondii*, this protein is a crucial motor for centrosome positioning and apicoplast inheritance [33]. Actin, which has been identified in all apicomplexans [34], was also identified in our results. Actin is crucial for segregation of the apicoplast and parasite maturation in *T. gondii* and *P. falciparum* [35,36]. Apicoplast inheritance is an actin-based process conserved across the phylum, and depletion of actin results in a strong apicoplast segregation defect in *T. gondii* and *P. falciparum* [35,36,37]. Additionally, actin is required for fusion of endocytic vesicles and vesicular transport in *P. falciparum* [38]. Furthermore, actin polymerizes into filaments (F-actin) to form static or highly dynamic networks [34]. In the present study, DEPPs in the SO7h vs. USO and S vs. SO comparisons were linked to actin cytoskeleton organization and actin filament bundle organization. Both actin and F-actin play important roles in the *E. tenella* life cycle, including invasion, gliding motility, egress, vesicular transport, apicoplast inheritance, intracellular replication, actin network maintenance, and material exchange between parasites [34].

Apicomplexans have a conserved endomembrane system that is efficient for protein trafficking, including the endocytosis and exocytosis needed for their obligate intracellular lifestyles [39]. There are three specialized apical secretory organelles, termed micronemes, rhoptries, and dense granules. During exocytosis, these secretory organelles release numerous protein complexes, such as adhesins, perforins, and proteases, which participate in many functions of parasites, including adhesion, escaping from the vacuole, gliding motility, and invasion [40,41]. Exocytosis of microneme proteins during invasion has been reported for *E. tenella* [42,43].

In the present study, two DEPPs (Rab5c and Rab3) associated with positive regulation of exocytosis were identified in the S vs. SO comparison. Rabs belong to the small G protein family and function as multifaceted organizers of membrane vesicular trafficking processes [44]. Recent studies confirmed a crucial role for Rab5C as a regulator in secretory organelle biogenesis [45]. Functional ablation of Rab5C leads to defective rhoptry biogenesis [46]. In *T. gondii*, Rab5c is a key regulator of the essential vesicular transport of microneme and rhoptry proteins [46]. Certain proteins require Rab5C for their routing towards the apical complex, such as rhoptries and micronemes [46]. Rac2 is an essential mediator for the maturation of pre-Golgi intermediates, which are involved in protein transport through the early secretory pathway [47,48]. In *P. falciparum*, Rab2 interacts with a glycolysis enzyme that mediates the transfer of cytosolic components to membranes [49]. In *T. brucei*, Rab2 is required for exocytosis, which mediates trafficking through the early secretory pathway [50].

Exocytosis is regulated in response to extrinsic stimuli and elevated cytosolic calcium levels [41]. Microneme exocytosis is correlated with intracellular Ca^2+^ levels in *T. gondii* [40]. In the present study, two DEPPs involved in calcium ion transport (*p* value = 6.17 × 10^−4^) were identified in sporozoites (P-type Ca^2+^-ATPase and calcium-transporting ATPase). It has been reported that Ca^2+^ fluxes provide signals to stimulate microneme secretion and gliding motility in parasites [39]. In *E. tenella*, acetaldehyde stimulates increased intracellular calcium levels, thereby triggering the secretion of microneme 2 proteins (EtMic 2) and promoting the motility of parasites. This process can be blocked by cellular calcium inhibitors, indicating that intracellular Ca^2+^ levels can impact the secretion of micronemal proteins in *E. tenella* [51].

## 4. Materials and Methods

### 4.1. Chickens and Parasites

Chickens (Lingnanhuang: LNH) were reared under standard hygienic conditions with free access to water and food (no anthelminthics or anticoccidial drugs). The experimental protocols were in accordance with the animal care guidelines and approved by the Ethics Committee of Lanzhou Veterinary Research Institute, Chinese Academy of Agricultural Sciences, China (No. LVRIAEC2014-001, 3 January 2014). Chickens at 2 weeks old were infected by oral dosing with 5 × 10^4^ sporulated oocysts. *E. tenella* (Guangdong Strain) oocysts were recovered from the ceca 7 days (168 h) later using techniques as described previously [52]. Partially sporulated (7 h) oocysts, were sporulated in vitro (28 °C, 120 rpm, 2.5% potassium dichromate (K_2_Cr_2_O_7_)) for 7 h. Completely sporulated oocysts were incubated (28 °C, 120 rpm) for 48–72 h in 2.5% K_2_Cr_2_O_7_. Sporozoites were isolated from cleaned oocysts by in vitro excystation and purification through cellulose columns of nylon wool and DE-52 [53].

### 4.2. Protein Extraction

Samples were first grinded by liquid nitrogen. Then samples (0.8 mL) were sonicated on ice using a high intensity ultrasonic processor (Scientz, Ningbo, China) in 4 volumes of phenol extraction buffer (containing 10 mM dithiothreitol (DTT), 1% protease inhibitor, and 1% phosphatase inhibitor). An equal volume of Tris-saturated phenol was added, and the mixture was centrifuged at 5500× *g* at 4 °C for 10 min. The upper phenol phase was transferred to a new centrifuge tube. Proteins were precipitated by adding at least 5 volumes of 0.1 M ammonium acetate/methanol and incubated overnight. After centrifugation at 4 °C for 10 min, the supernatant was discarded. The remaining precipitate was washed with 3 mL of methanol once, followed by 3 mL of acetone twice. Finally, the protein was redissolved in 1.5 mL of 8 M urea and the protein concentration was determined with a BCA kit (Beyotime, Shanghai, China) according to the manufacturer’s instructions.

### 4.3. Trypsin Digestion

The final concentration of 20% TCA was slowly added into the proteins, the mixture was vortex mixed, then precipitated at 4 °C for 2 h. After centrifugation (4 °C, 5 min, 4500× *g*), the supernatant was discarded, and the remaining precipitate was washed with 3 mL of pre-cooled acetone 2–3 times. The protein sample was then diluted by adding 3 mL of 200 mM TEAB. The protein solution was reduced with dithiothreitol (DTT) (final concentration 5 mM) for 30 min at 56 °C and alkylated with iodoacetamide (IAA) (final concentration 11 mM) for 15 min at room temperature in darkness. Trypsin was added in a ratio of 1:50 (protease:protein, *m/m*) for digestion overnight.

### 4.4. Affinity Enrichment

The peptide mixtures were dissolved in the loading buffer solution (50% acetonitrile/6% trifluoroacetic acid), ensuring that the concentration of peptides was 3 mg/mL. Then, the supernatant was transferred to the pre-washed IMAC (immobilized metal affinity chromatograph) material (ThermoFisher Scientific, Waltham, MA, USA, Catalog Number A32992), which was placed on a rotating shaker and gently shaken for incubation. After incubation, the IMAC resin was washed with 50% acetonitrile/6% trifluoroacetic acid and 30% acetonitrile/0.1% trifluoroacetic acid, sequentially. To elute the enriched phosphopeptides from the IMAC resin, elution buffer containing 10% NH_4_OH was added, and the eluate was collected and lyophilized. For LC-MS/MS analysis, the resulting peptides were desalted with C18 ZipTips (Merck Millipore, Billerica, MA, USA) according to the manufacturer’s instructions.

### 4.5. LC-MS/MS Analysis

LC-MS/MS analysis was performed with a timsTOF Pro mass spectrometer (Bruker Daltonics, Hamburg, Germany) combined with nanoElute UHPLC (Bruker Daltonics, Hamburg, Germany). For the phosphoproteomic experiments, enriched phosphopeptides samples were dissolved in mobile phase A (0.1% (*v/v*) formic acid) and loaded onto a home-made reversed-phase analytical column (15 cm length, 75 μm id). Mobile phase B was an acetonitrile (ACN) solution containing 0.1% formic acid (FA). The gradient of the LC was set as: 2% to 22% buffer B (0–50 min), 22% to 35% B (50–52 min), 35% to 90% B (52–55 min), and 90% B (55–60 min). The flow rate was set to 0.45 µL/min. After the separation, the peptides were injected into a capillary ion source for ionization and analyzed by the timsTOF Pro mass spectrometer. The ion source voltage was set at 1.6 kV, and the peptide parent ions and their secondary fragments were detected and analyzed using TOF. The scanning range for secondary mass spectrometry was set to 100–1700 m/z. The data acquisition mode was the parallel cumulative serial fragmentation (PASEF) mode. After first-order mass spectrometry collection, the second-order spectrogram with the charge number of parent ions in the range of 0–5 was collected in PASEF mode 10 times. The dynamic exclusion time of tandem mass spectrometry scanning was set to 30 s to avoid repeated scanning of parent ions.

### 4.6. Database Search

The resulting MS/MS data were processed using the MaxQuant search engine (v.1.6.6.0, https://maxquant.net/maxquant/, accessed on 15 August 2020). Tandem mass spectra were searched against the *Eimeria_tenella*_5802_UP_20200108 database (9323 sequences) concatenated with a reverse decoy database. Trypsin/P was specified as a cleavage enzyme allowing up to 2 missing cleavages. A common contamination database was added to eliminate the influence of contaminated proteins in the identification results. The minimum peptide length was set to 7 amino acid residues and the maximum number of peptide modifications was set to 5. The mass tolerance for precursor ions was set as 20 ppm in the first search and 20 ppm in the main search, and the mass tolerance for fragment ions was set as 0.02 Da. Carbamidomethyl on Cys was specified as a fixed modification, and acetylation modification, oxidation on Met and phosphorylation modification were specified as variable modifications. FDR was adjusted to <1%. The mass spectrometry data have been deposited to the ProteomeXchange Consortium via the PRIDE [54] partner repository with the dataset identifier PXD029545.

### 4.7. Bioinformatics Analysis

The Gene Ontology (GO) [55] annotation was derived from the UniProt-GOA [56] database (http://www.ebi.ac.uk/GOA/, accessed on 25 August 2020). Firstly, identified protein IDs were converted to UniProt [57] (http://www.uniprot.org, accessed on 25 August 2020) IDs and then mapped to GO IDs. If some identified proteins were not annotated by the UniProt-GOA database, InterProScan of InterPro [58] (http://www.ebi.ac.uk/interpro/, accessed on 25 August 2020) was used to annotate the GO functional protein based on the protein sequence alignment method. Then, proteins were classified by Gene Ontology annotation based on three categories: biological process, cellular component, and molecular function. DEPPs were classified according to GO annotations and Kyoto Encyclopedia of Genes and Genomes (KEGG) [59] pathways (http://www.genome.jp/kegg/, accessed on 26 August 2020). A two-tailed Fisher’s exact test was employed to test the enrichment of the DEPPs against all identified proteins, and *p* value < 0.05 was the significance criterion. The interactions among DEPPs were analyzed using the STRING (Search Tool for the Retrieval of Interacting Genes/Proteins) [60] database (http://string-db.org/, accessed on 27 August 2020). The confidence score was set at the medium level (≥0.400). Cytoscape (v3.7.1) [61] was used to visualize protein–protein interaction networks. The MCODE (the molecular complex detection) [62] plug-in in Cytoscape was used to analyze highly connected nodes and functional modules of PPI with default parameters. TCseq (v1.16.0) [63] in the R package was used to conduct the clustering analysis of proteins.

## 5. Conclusions

We performed a large-scale analysis of quantitative phosphoproteomic differences between unsporulated oocysts, partially sporulated oocysts, sporulated oocysts, and sporozoites of *E. tenella*. DEPPs were linked to multiple biological processes. Cluster and network analyses were also performed to clarify the mechanisms operating in the *E. tenella* life cycle. Our results provide important resources for further research and shed new light on the regulation of protein post-translational phosphorylation during *E. tenella* development.

## Data Availability

Data are available upon request from the corresponding author or via ProteomeXchange with identifier PXD029545.
